# Parastomal gallbladder herniation: A case report and review of the literature

**DOI:** 10.1016/j.ijscr.2020.07.002

**Published:** 2020-07-26

**Authors:** Beat Moeckli, Perparim Limani, Pierre-Alain Clavien, Rene Vonlanthen

**Affiliations:** a Department of Surgery and Transplantation, University Hospital Zurich, Raemistrasse 100, CH-8091, Zurich, Switzerland; b Department of Surgery, University Hospital Geneva, Rue Gabrielle-Perret-Gentil 4, 1205, Genève, Switzerland

**Keywords:** PSH, parastomal hernia, CT, computed tomography, COPD, chronic-obstructive lung disease, ASA, American Society of Anaesthesiology, CRP, C-reactive protein, PCT, procalcitonin, Case report, Enterostomy, Parastomal hernia, Gallbladder herniation, Gallbladder incarceration

## Abstract

**Introduction:**

Parastomal hernia is a common complication of patients living with an enterostomy. However, a parastomal hernia involving the gallbladder is a rare condition with only eight cases documented in the literature.

**Presentation of case:**

We report the case of a 69-year old female who underwent an open right hemicolectomy with creation of a colostomy and terminal ileostomy. She presented with parastomal swelling and pain 16 months later. A computed tomography scan revealed a parastomal herniation of the gallbladder. We elected to proceed with a cholecystectomy and hernia repair, the patient was asymptomatic at her last follow-up.

**Discussion:**

A systematic search of the literature found eight previously published cases. This condition primarily affects elderly females. Five patients were treated surgically and three conservatively, all with a favorable outcome. In frail patients without complicating factors, a conservative treatment approach with surveillance may be safe. We chose a surgical approach due to the symptomatic nature of the presentation and the gallstone containing hernia. This is the first case of a parastomal gallbladder herniation containing a large gallstone.

**Conclusion:**

This report should help broadening the physician’s differential diagnosis in dealing with patients with symptomatic parastomal hernias and provide an example for diagnosis and management of this complication.

## Highlights

• Gallbladder herniation is a rare complication of a parastomal hernia, affecting primarily elderly females.
• For patients with parastomal swelling and pain gallbladder herniation should be included in the differential diagnosis.
• Elderly patients with multiple comorbidities may benefit from a conservative approach instead of surgery.
• The use of prophylactic mesh when creating a permanent end colostomy reduces the rate of parastomal hernias.

## 1 Background

Enterostomy placement is one of the most common surgical procedures worldwide. In Switzerland, about 1200 new stomas are created annually and 1000 patients per million inhabitants live with a stoma [1]. Development of a parastomal hernia (PSH) is a common problem after a definitive enterostomy creation. PSH is defined as a type of incisional hernia arising through a fascial defect between the enterostomy and the surrounding soft tissue [2]. The passage of the intestine through the abdominal wall represents a weakness facilitating the hernia formation. The reported incidence of PSH ranges up to 56% in patients undergoing a colostomy creation [3]. Asymptomatic patients might qualify for conservative treatment with abdominal binders or ostomy belts. However, in 11–70% of patients, surgical repair is performed because patients are symptomatic or to prevent complications and potentially emergency surgery. Unfortunately, parastomal hernia repair is associated with a high recurrence rate [4].

Most PSH involve a reducible segment of the omentum, colon, or small bowel, more rarely are cases involving herniation of the stomach [5]. In contrast, PSHs involving herniation of the gallbladder are unusual. We herein report a case of PSH with gallbladder herniation diagnosed by CT and treated with an open cholecystectomy and direct hernia repair and discuss its management. This work has been reported in line with the surgical case report (SCARE) guidelines criteria [6].

## 2 Case presentation

A 69-year-old female patient with a past medical history of hypertension and coronary heart disease as well as corticosteroid-treated chronic obstructive lung disease and a Body Mass Index of 25 kg/m2 underwent an urgent explorative laparotomy with right hemicolectomy and ileotransversostomy due to ischemic cecal necrosis. We performed a relaparotomy with creation of a colostomy and terminal ileostomy due to an anastomotic leakage on postoperative day six after the initial operation.

After an uneventful postoperative rehabilitation, the patient presented to the outpatient clinic 16 months after the initial operation with parastomal swelling, tenderness and pain. The physical exam revealed a parastomal hernia under Valsalva in the erect position and an asymptomatic incisional hernia of the former median laparotomy.

**Fig. 1:**
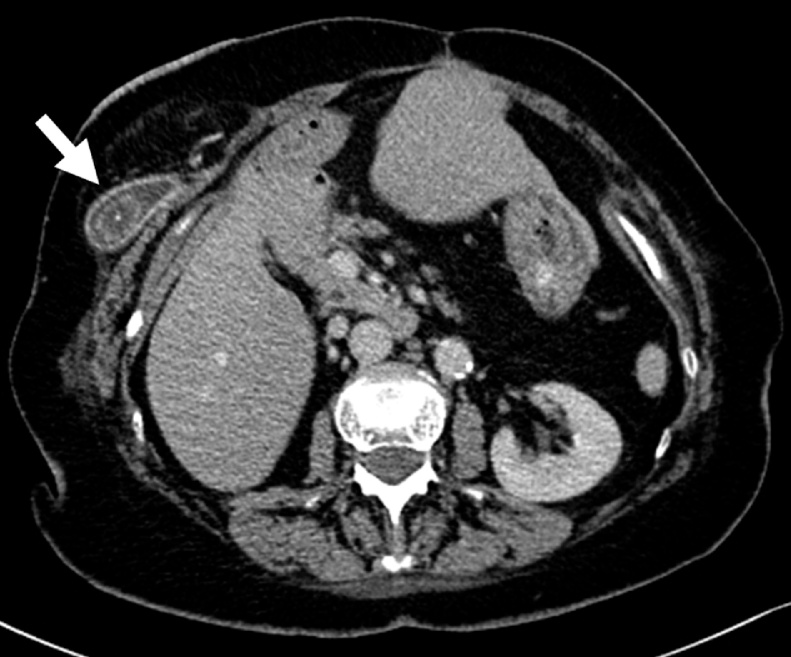
**Abdominal CT scan**. Gallbladder herniated into the parastomal hernia (arrow).

Due to the symptomatic nature of the parastomal hernia, we decided to perform an abdominal computed tomography scan (CT) (Fig. 1). The exam revealed a subcutaneous herniation of the gallbladder into the parastomal hernia defect with cholelithiasis (one stone with a diameter of 2 cm) but no radiographic findings of cholecystitis. Inflammatory markers were increased (C-reactive protein (CRP) 22 mg/l, procalcitonin (PCT) 0.12 μg/l), with a leukocyte count of 18’560/μl displaying a neutrophilic shift. Regarding the nutritional state, the levels of total protein, albumin, prealbumin, and selenium were decreased. Heart, liver, kidney, and pancreas laboratory tests were normal. Urinalysis revealed a urinary tract infection.

Our clinical suspicion of a parastomal hernia was substantiated through the results of the CT scan. The finding of a gallbladder herniation was a surprise but the preoperative diagnosis helped with the planning of the procedure.

**Fig. 2:**
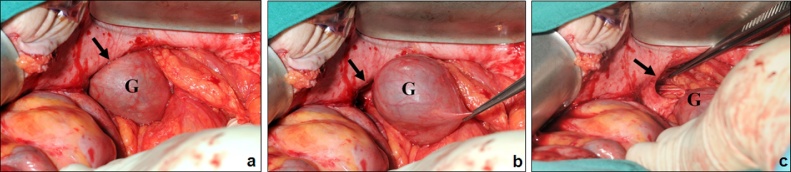
**Intraoperative Findings.** a) Gallbladder (G) within the parastomal hernia (arrow). b) Reduction of the gallbladder (G) out of the parastomal hernia (arrow) c) Parastomal hernia orifice (arrow) after reduction of the gallbladder (G).

After elective laparotomy, the gallbladder was carefully reduced out of the parastomal hernia (Fig. 2) followed by retrograde cholecystectomy. The ostomy was then reversed with a side-to-side ileo-colic anastomosis. The parastomal and the median incisional fascial defects were both closed with a continuous nonabsorbable suture. Histological findings of the gallbladder showed a low-grade chronic cholecystitis with reactive epithelial malformation and gastric metaplasia. The postoperative course was complicated by a wound infection that was treated with a Vacuum Assisted Closure (V.A.C.®) therapy, but was otherwise unremarkable. We discharged the patient 34 days after surgery. At the last follow-up visit four years postoperatively, the patient presented with an asymptomatic recurrent small incisional hernia at the median laparotomy site that we treated conservatively. The patient has since died from a cardiac event unrelated to the condition described in this case report.

## 3 Discussion

Parastomal herniation of the gallbladder is uncommon. We conducted a systematic search of the EMBASE and MEDLINE databases with the following terms ‘gallbladder’ and ‘parastomal hernia’. We found 12 published articles in this search of which we included seven in this article. One additional manuscript was identified through bibliographic analysis. In total, we identified eight other cases of parastomal gallbladder herniation (Table 1) [[7], [8], [9], [10], [11], [12], [13], [14]]. The average age of the previously described cases is 77 years with 75% of patients being female. The older age at presentation is not surprising as decreased connective tissue elasticity is associated with elderly age. An unusually long gallbladder mesentery or atrophy of the liver are also risk factors for a mobile gallbladder. The patient demographic and the disease process involved thereby resembles the one of gallbladder torsion [15]. Our case is the first described in the literature with the presence of a gallstone in the hernia. With one out of nine presentations, cholelithiasis does not appear to be a risk factor for parastomal gallbladder herniation but may increase the risk for incarceration and cholecystitis and may therefore favor a surgical approach [16]. Several risk factors for developing a PSH have been described [5]. Of these risk factors, poor nutritional status, increased intra-abdominal pressure, as well as corticosteroid therapy due to COPD were present in our patient.

**Table 1:** Summary of previously published cases of parastomal herniation of the gallbladder.

	Year	Age	Sex	Diagnostic modality	Presentation	Treatment	Outcome
Rogers et al. [7]	2019	75	F	CT	Symptomatic, increasing abdominal pain, incarcerated hernia	Surgical, no cholecystectomy but reinforcement of PSH with synthetic on-lay mesh	Discharge on POD 5 no complications at 6 months
Brown et al. [8]	2018	63	F	CT	Asymptomatic, painless parastomal bulge	Surgical, ostomy takedown, cholecystectomy, abdominal wall reconstruction with on-lay bio prosthetic mesh	No recurrence at follow-up
Bakshi et al. [9]	2017	89	M	CT	Symptomatic, burning around ostomy and small bowel obstruction	Conservative due to comorbidities, antibiotics, nasogastric tube	Discharge on hospital day 5
Frankl et al. [10]	2017	88	F	CT	Symptomatic, fever, abdominal pain	Conservative due to comorbidities, antibiotics, nasogastric tube	Discharge on hospital day 7
To et al. [11]	2015	85	F	CT	Symptomatic, increasing pain at ileal conduit	Surgical, open cholecystectomy without closure of hernia defect	Discharge POD 5, well at 1 month visit
Rosenblum et al. [12]	2013	76	M	CT	Symptomatic, abdominal pain	Surgical, open cholecystectomy	Discharge POD 5
Garcia et al. [13]	2005	63	F	CT	Symptomatic, nausea, abdominal pain	Conservative due to comorbidities	Discharge on hospital day 2, asymptomatic at 16 months
St Peter et al. [14]	2005	73	F	Surgical exploration	Symptomatic, abdominal pain, hernia incarceration	Surgical, open cholecystectomy without closure of hernia	NA

F, Female; M, Male; PSH, Parastomal hernia; CT, computed tomography scan; NA, not available; POD, postoperative day.

Of the previously described cases, three were successfully treated conservatively despite an initially symptomatic presentation [9,10,13]. Even though in all three patients conservative management was attempted because of significant comorbidities, their favorable outcome indicates that a conservative approach may be valid if the gallbladder is not incarcerated and the patient is hemodynamically stable at presentation [3]. This approach needs to be constantly reappraised to detect complications such as emphysematous cholecystitis as described by Rosenblum et al. [12] No matter the presentation, a surgeon needs to carefully balance the risks of PSH repair with the risks of conservative treatment such as incarceration, intestinal obstruction, or hollow organ perforation. The described condition mostly affects elderly patients, often with several comorbidities and to integrate their perspective is essential. We chose a surgical approach due to the symptomatic presentation of our patient and the presence of a large gallstone located in the gallbladder fundus.

The high incidence of PSH with its subsequent adverse effects on quality of life, the need for surgical repair, and increased cost provide the reason to prevent PSH formation rather than treat its consequences [17]. In accordance with the convincing results of using prosthetic mesh for incisional hernia repair and international guidelines, it seems reasonable to insert a prophylactic prosthetic mesh during the creation of the ostomy in order to prevent PSH formation [3,18]. However, prosthetic meshes must be used in close-to-sterile situations, because infections of prosthetic material are the cause of major complications. Unfortunately, in the case presented here, the ostomy was created in the context of an anastomotic bowel leak with a contaminated setting. New mesh types have been developed during recent years to minimize the risk of infection. For example, bio prosthetic meshes and lightweight meshes with partially absorbable components, both demonstrated clinical safety. This may in the future extend the preventive options [19].

Diagnostic tools including clinical (e.g. Valsalva maneuver) as well as radiological methods such as ultrasound and CT have been reported for the diagnosis of PSH [20]. Most previously reported cases used CT scan for the diagnosis of the parastomal hernia, independent of the presentation. However, in asymptomatic patients we advocate relying on clinical examination or ultrasound in a first step to allow for a dynamic assessment and avoid cost and radiation associated with a CT scan. If there is a doubt about the structures involved in the herniation after the clinical examination or in a symptomatic patient, the physical exam needs to be complemented by a CT scan. Knowledge of the involved anatomical structures as well as the extent of the PSH is crucial to prevent unexpected findings during surgery.

## 4 Conclusion

Our report and the summary of the previous literature may promote the physician's awareness for a differential diagnosis in elderly females with symptomatic PSH, increase the understanding of this rare condition and guide its diagnosis and treatment.

## Conflicts of interest

There are no actual or potential conflicts of interest capable of influencing judgment on the part of any author.

## Funding

This research did not receive any specific grant from funding agencies in the public, commercial, or not-for-profit sectors.

## Ethical approval

This retrospective case report is exempt from ethical approval at our institution.

## Consent

Written informed consent was obtained from the patient’s son for publication of this case report and accompanying images. A copy of the written consent is available for review by the Editor-in-Chief of this journal.

## Author contribution

BM, PL, PAC, RV were involved in the conception of the work.

PL and RV were involved in the surgical treatment of the patient.

BM and PL drafted the manuscript, RV and PAC critically revised the manuscript.

All authors read and approved the final manuscript.

## Registration of research studies

This is not human subjects research.

## Guarantor

Beat Moeckli, MD.

## Provenance and peer review

Not commissioned, externally peer-reviewed.
